# Critical realism: a practical ontology to explain the complexities of smoking and tobacco control in different resource settings

**DOI:** 10.3402/gha.v6i0.19303

**Published:** 2013-04-03

**Authors:** Dunsi Oladele, Alexander M. Clark, Solina Richter, Lory Laing

**Affiliations:** 1 School of Public Health, University of Alberta, Edmonton, Alberta, Canada; 2 Faculty of Nursing, University of Alberta, Edmonton, Alberta, Canada

**Keywords:** critical realism, smoking, developing countries, Africa, Nigeria, Lagos, health policy, tobacco control

## Abstract

**Background:**

This paper presents critical realism (CR) as an innovative system for research in tobacco prevention and control. CR argues that underlying mechanisms are considered and explored to ensure effective implementation of any program/policy or intervention. Any intervention or program/policy that is transposed from one country to another or one setting to another is complex.

**Methods:**

The research was undertaken and analyzed through a critical ethnography lens using CR as a philosophical underpinning. The study relied upon the following components: original fieldwork in Nigeria including participant observation of smokers, in-depth interviews and focus groups with smokers, and in-depth interviews with health professionals working in the area of tobacco control in Nigeria.

**Results:**

Findings from this small ethnographic study in Nigeria, suggest that Critical Realism holds promise for addressing underlying mechanism that links complex influences on smoking.

**Conclusion:**

This paper argues that understanding the underlying mechanisms associated with smoking in different societies will enable a platform for effective implementation of tobacco control policies that work in various settings.

In the early 1950s, cigarette smoking became widely recognized among public health experts as a cause of morbidity and mortality. Although physicians and researchers had speculated in the early part of the 20th century as to the possible relationship between smoking and various cancers, they had no conclusive scientific support for their hypothesis. In the same decade, several published studies highlighted the dangers of smoking by providing powerful empirical evidence linking smoking to the development of lung disease, as well as other respiratory problems in humans (1). Based on the results of these studies, in 1954, the Canadian Medical Association (CMA) issued the first public warning on the hazards of smoking, and, in 1961, concluded that cigarette smoking caused lung cancer (1). In 1962, the Royal College of Physicians of London also declared that: ‘cigarette smoking is a cause of lung cancer and bronchitis, and probably contributes to the development of coronary heart disease’ (1). Similarly, in 1964 the United States (US) Surgeon General's report on smoking and health alerted the nation to the health risks of smoking (2).

The effect of publicizing this knowledge initiated the beginning of smoking cessation initiatives in North America and other Western countries. Concerted efforts were made by public health policy makers and practitioners both to help smokers quit and to dissuade non-smokers from starting the habit (1). Many people still choose to smoke, in spite of the warnings about the associated health risks, the initiatives to discourage smoking commencement, smoking cessation support systems, and restrictions on the behavior itself. Admittedly, the combination of these initiatives has led to a substantial reduction in smoking (3). However, recent estimates indicate the prevalence of 6 million smokers in Canada (Health Canada, 2009) and 46 million smokers in the United States (4). It appears that smokers who were willing to quit have done so; therefore, what appears to remain are the many smokers who do not want to or simply find it difficult to quit.

The cigarette consumption rate in Africa is on the rise. In 1995, the total annual cigarette consumption in the region was 131,181 million cigarettes. This increased to 212,788 million in 2000 (5). The strong and compelling evidence from research on the adverse health effects of cigarette smoking led the World Health Organization (WHO) to take steps toward regulating tobacco production, consumption, and advertising. One particularly significant initiative was the WHO's directive to the World Health Assembly (WHA), an arm of the WHO, to create the Framework Convention on the Control of Tobacco (FCTC) in May 2003 (6). The many governments that signed and ratified the FCTC initiative perceive this initiative as an international instrument for controlling tobacco manufacturing, marketing, and distribution. The FCTC directly addresses various aspects of the tobacco trade, including but not limited to: advertising, promotion and sponsorship, taxation, labeling, product content, passive smoking, smuggling, smoking cessation, and so forth (7).

This article addresses the importance of utilizing critical realism (CR) to understand smoking and tobacco control in different resource settings, with particular emphasis and exposition of the situation in Nigeria. Formally known as the Federal Republic of Nigeria, the country is located in West Africa. Nigeria has an area of 923,768 km^2^, including about 13,000 km^2^ of water. It has an estimated population of 150 million. The official language is English, and other widely used main languages are Yoruba, Igbo, and Hausa. Nigeria's literacy rate is 68% on average with a higher rate for men (75.7%) and (60.6%) for women. Nigeria's economy is struggling to leverage the country's vast oil wealth in order to alleviate the poverty that affects 57% of the population. Economists refer to the co-existence of vast natural resources and extreme poverty in developing countries like Nigeria as the ‘paradox of plenty’ or specifically in the Nigerian case as ‘the curse of oil’. The poor condition of health and appalling health care delivery system in Nigeria are the primary factors responsible for the low life expectancy of 47 years (8).

## Methods and materials

I, the researcher (first author), interviewed a total of 42 individuals consisting of active smokers and health professionals in Lagos Nigeria. Twenty smokers participated in in-depth interviews, while another group of 15 smokers participated in a focus group. In addition, seven health professionals working in tobacco control were interviewed. After interviewing 30 participants, key categories in the analysis started to emerge. Ethics approval for this study was obtained from the ethics board of the Lagos University Teaching Hospital in Nigeria and the University of Alberta in Canada.

### Sampling

A purposive sampling method was used for selecting smokers for the interviews and focus groups. Subjects were selected based on the fact that they currently smoke cigarettes. The convenience sampling method was used for selecting the health professionals for the interviews. Recruitment of subjects occurred mostly through word of mouth from other smokers’ interviewed, distribution of a study introduction letter, and referrals for health professionals’ interviews.

### Inclusion criteria

The following inclusion criteria were used for the selection of study participants. Current smokers, male or female, over the age of 18 years who were willing to take part and give informed consent; who have smoked every day or almost every day for the last year; who are not currently in a smoking cessation program, are not using smoking cessation aids, are not making a concerted effort to quit smoking, or are not expecting to quit within the next year; and who are willing to participate in a 1 hour interview or a 2 hour focus group session.

## Data collection strategies

1. **Participant observation** – The research design was basically to describe the experiences of smokers as much as possible. I acted as a complete observer (the complete observer is a covert observer, i.e. not interacting with people) of smokers in Nigeria in places like bars, markets, residential, and office areas. In this role, I observed smokers on a daily basis for 1 month and wrote detailed field notes of my observations. At the beginning of the second month, I took on the role of an observer as a participant. In this role, I interacted with smokers, interviewed them on why they smoke, the role smoking plays in their lives, and what can make them stop smoking. I went to different parts of Lagos, Nigeria, including the upper- and lower-class neighborhoods to observe smokers. I started out by going out every day to observe smokers in different neighborhoods but soon found out that many Nigerians are closet smokers, i.e. they do not smoke in public places. The few people I found smoking in public places were personal chauffeurs and bus and taxi drivers. The only public places where I found people smoking were mostly in restaurants and in bars. As a result, I realized that substantial information on smoking was not collected in Nigeria through participant observation. The smokers I observed usually come out to smoke around lunchtime, and they do it in groups, i.e. smoking and conversing. In the restaurants and bars, smoking is also a group/social activity as people smoke, eat, drink, and have friendly conversations. I observed that Nigerian smokers are social smokers; they like to smoke together, while eating, talking, or drinking.
2. **In-depth interviews of smokers** – I performed individual in-depth interviews with 20 smokers and conducted three focus groups each composed of five people (see Appendix A). This started 1 month after the observation of smokers in different parts of Lagos, Nigeria. The objective of qualitative research sampling is to understand the phenomenon of interest as opposed to putting the major focus on sampling. I used the purposive sampling method where subjects were selected based on the fact that the individual smokes cigarettes. The smokers are the ones that can give me the best information on why they continue to smoke. After 1 month of observation in the different parts of Lagos, I distributed a letter of introduction for the research (see Appendix D) in the different neighborhoods. This letter included my contact telephone number and address, the location of the interviews and the focus groups, information on the research, and the inclusion criteria for interviews and the focus groups. When interested participants called, I introduced myself as a researcher, and I then asked the inclusion criteria questions before proceeding to invite the respondent for the interviews. If, on the other hand, the smoker did not qualify to participate in my study, I thanked the smoker and explained why I could not interview the smoker based on the information given to me by the smoker. On the day of the interview, if the smoker is willing and able to participate, I meet the smoker at a place we have both mutually agreed upon to meet. I always make sure this is a public place because of the safety issues in Nigeria. The consent form was usually read out and given to the smoker to read and sign to indicate willingness to participate in the study. The interviews were audio taped and lasted on average for 30 min per respondent. The interviews and focus groups were done over a period of 4 weeks. Smokers were mainly recruited by me, the researcher (first author), by personally visiting the bars and eating joints in Lagos. I walk up to any individual I have watched smoking for a couple of minutes in the bar or eating joint, introduce myself as a researcher and asked if the smoker is willing to participate in a 30 min interview on smoking in Nigeria. If the smoker answered yes, I bring out the consent form and explain the study and the interview process to the smoker. The respondents were also offered refreshments and allowed to smoke during the interview. I made sure that I had about 10 interviews per week and went out to observe smokers in restaurants. The smokers’ ages ranged from 22 to 41 years. Five female smokers were interviewed and four from high socio-economic status. The rest of the smokers were mainly from the middle- and lower-class group.
3. **In**-**depth interviews of health professionals in Nigeria** – In addition, I conducted seven in-depth interviews with health professionals in the tobacco control policy sector in Nigeria. The health professionals included three medical doctors, one tobacco litigation lawyer, and three others working in tobacco control in Nigeria. I am cautious not to describe their background fully as they can all be easily identified in Nigeria. The questions, which were in an open-ended format, covered what the policy makers in the area of health and tobacco in Nigeria were putting forward as what works and what does not work in reducing the number of people who smoke (see Appendix B). The in-depth interviews entailed booking an appointment ahead of time and meeting with the seven key informants who are all experts in their various fields and currently working to reduce smoking in Nigeria. The informants were interviewed individually at different times as identified from referrals. Three of the health professionals were referred by some of the other health professionals I interviewed previously. A convenience sampling of seven individuals was used. Some interviews lasted an hour and some more than 1 hour and all the interviews were audio taped with the consent of the respondents. Questions were based primarily on what will work to reduce the rate of smoking in Nigeria and who the major political players in Nigeria are with respect to health and smoking.
4. **Focus group (FG)** – The focus group was an attempt at triangulation. For example, in this study, different data methods were used on the field such as field notes, participant observation, focus group, and interviews. The rationale for triangulation is that it attempts to overcome any weakness or bias of a single research strategy (9). A focus group is a qualitative research tool in which a group of people are asked about their attitudes toward a product, service, concept, ideas, advertisement or packaging. Questions were asked in an interactive group setting where participants are free to talk with other participants. The focus group allowed me to gather experiences and beliefs of participants. Three focus groups were conducted; each consisting of five people (all males) with an age range of 22–33 years. Topics were based on perceived benefits of smoking, what smoking means to them (their attitudes, opinions, and perceptions), the role smoking plays in their lives, perceptions of policies regarding smoking, knowledge of the risks of smoking. The focus was on people who currently smoke and those not planning to quit (see Appendix A). The focus group participants were asked to complete a brief demographic and smoking behavior background questionnaire (see Appendix C) that functioned to focus their attention on the topic of discussion. The focus groups were formed in a bar or eatery after obtaining consent from smokers who were at the bar on that day. Guiding questions designed to target the topic of interest were used to initiate the discussion. The focus group and interviews were conducted in English (English is the official language used in teaching and business in Nigeria). Pidgin English was not used in any of the interviews. Pidgin English is a local patois based on the English language that is used for trade or communication between people from different Nigerian tribes who speak different languages.

## Data analysis

I, the researcher, used the services of a professional transcriber, and I read and re-read the collected data several times. I highlighted sections in the text and made comments in the margins on anything that I found striking. Highlighted sections of the text were grouped into categories. Sub-categories that emerged were created. The sub-categories were judged based on the following criteria:

1. Do the data reflect the category and fit nicely into it?
2. Do the category make sense?
3. How are the categories related?
4. What main patterns keep recurring in the data?
5. What conclusions can be drawn?

Common themes that ran through the data were identified, and themes were compared in each transcribed note. Commonalities and differences were noted, and to conclude, I identified the overall themes that best describe the experiences and responses of participants (10–13).

### 

#### Smoking and tobacco control in CR terms

Tobacco control policies have resulted in an overall reduction in the prevalence of smoking in several developed countries (4, 14). These gains, however, have come with associated problems, such as the creation of smoking inequalities between not only the rich and the poor within a country, but also, smoking inequalities between the rich and the poor countries (15–18). When smoking decreased in the West and the Western public health services were rejoicing, the unintended consequence was that smoking prevalence increased among the poor in the West and increased in Africa and other developing countries. The reasons for this phenomenon remain unclear. According to Bobak et al. (2000), the success of smoking reduction strategies depends, in part, on a better explanation of these phenomena (15). However, a noteworthy fact is the decrease in cigarette smoking observed in the West for a certain period, in that smoking rates appear to have stalled in recent times. For instance, there was no observed change in US adult smoking rates from 2004 to 2006 (19). In Canada, from 2000 to 2006, the Canadian Tobacco Use Monitoring Survey (CTUMS) indicated that smoking prevalence is declining in smaller increments (20).

CR presents the bridge between theory and practice and appears to be a useful ontology in explaining the inequalities that exist in smoking within countries between different socio-economic groups and between more and less developed countries. The reason for this is that CR attempts to respond to and understand underlying mechanisms. The logic of CR lies in its attention to social and structural mechanisms, and the findings from this small ethnographic study suggest that CR holds promise in the tobacco control arena. The tobacco epidemic reflects a complex system of factors, processes, and mechanisms that take precedence over disciplinary, methodological or ideological predisposition. In the real domain, CR views behavior as being influenced by both agency and structural factors. Although humans constitute agency, agency is always constrained by wider structural factors that are viewed as surrounding the individual (21). For example, while culture is conceived as being dependent on and created only through the existence of humans, CR argues that culture exists independent of individuals. Likewise, social phenomena are made possible by the presence of humans but are deemed to be external to individuals and have existence and the power to constrain whether this is recognized by individuals or not (22–24).

It is notable to take the early works of Bhaskar into consideration for an auspicious beginning of what successful tobacco control policy will encompass using CR as a framework. The early work of Bhaskar conceptualized the existence of three realms of reality: the *actual*, the *real*, and the *empirical*. The *actual* domain refers to events and outcomes that occur in the world. The *real* domain refers to underlying relations, structures, and tendencies that have power to cause changes in the actual realm. The *empirical* dimension on the other hand refers to human perspective on the world (actual and real domains). This could be the perspective of an individual or a scientific inquiry. Most often these causal influences remain latent; however, under the right circumstances, factors in the real domain can act together to generate causal changes in the actual domain (21).

CR is positioned between positivism/objectivism and constructivism/relativism and recognizes the existence of knowledge independent of humans and, at the same time, recognizes the socially embedded and fallible nature of scientific inquiry. CR recognizes that there is no universal or one-size-fits-all solution to a problem and that problems are complex and multilayered. The challenges and problems of smoking in the West are different from the challenges and problems of smoking in Nigeria or other parts of Africa, due to differences in social, economic, and political systems. For instance, some advertisements in Nigeria still present smoking as socially desirable and glamorous, by showing young happy people engaged in the habit. Other advertisements relate smoking to dreamlike promises of prestige, power, freedom, luxury, and success (25). Young people see their role models, film stars, and musicians in smoking advertisements. This type of promotion leads to the unintended consequence of making a relational nexus between the act of smoking and the dreamlike promises of prestige, power, freedom, luxury, and success for the Nigerian youths.

One of the crucial and significant phrases in CR is the ‘model of generative causation’. Generative causation is not simple or linear because it takes into account numerous and multi-factorial aspect of what determines an effective policy/intervention/outcome. A change in one factor in an event can cause a big change in outcome. A good example used by Pawson and Tilley to explain this is the gunpowder illustration. In the book, *Realistic Evaluation*, Pawson and Tilley (1997) explain that gunpowder does not always ignite when the flame is applied. The causal connection involved is thus not established by constant conjunction, nor is it a ‘humean’ perception of the mind. They explain that spark causes the explosion because of the chemical composition of the gunpowder and that chemists would explain the chemical composition as follows: it is a molecular structure of the mixture of potassium nitrate, charcoal and sulfur having the capacity to produce exothermic reactions. However, if the mixture is damp, if there is no oxygen present, if there is insufficient powder, and if the conditions are not right, there will be no explosions (26, p. 58).

The problems and understanding of smoking is multi-factorial. There is generative causation, i.e. many factors coming together to influence a particular phenomenon as opposed to explaining phenomenon through a linear causation as is often done through the successionist approach (a+b=c). In CR terms, rather than follow linear reasoning, attention is paid to mechanisms. There are a multitude of factors that affect change at the individual, family, community, and political level that come together to influence health and smoking in different societies.

What has consistently happened in the past in public health using the successionist model is that successes recorded in one region are believed and assumed to automatically work in another region without actually looking at specific peculiarities and differences of each region. For example, policies formulated in the West (like the tobacco control policy) are transposed to Africa and the policies are expected to work as effectively as they worked in the West. Developing countries are not as well poised as developed countries because they do not have the resources of the developed countries. The political climate in many developing countries is totalitarian or a ‘farce democracy’, as opposed to a true democracy, in which democratic ideology is upheld. Many failed health policies have followed a similar pattern – the absence of factoring in specific contexts, when policies are being implemented (27).

In contrast to the common view in the field of public health, CR shows causation is not linear in the sense that event A must cause event B if A precedes B regularly. This approach infers causation from regular sequences of events. Rather, CR views event as products of many factors coming together in certain combinations and given the right circumstances or context to causally generate new events (23). According to Sayer, what causes something has nothing to do with the number of times we have observed it happening. Explanation depends instead on identifying causal mechanisms and how they work, and discovering if they have been activated and under what conditions (28). Pawson and Tilley further asserted that, an action is causal only if its outcome is triggered by mechanisms acting in context. This bit of popular science is useful to us in that it contains all the ingredients of a realist causal proposition. Our basic concern is still, of course, the outcome (the spark causing the explosion). The explanatory work is first of all the mechanism (the chemical composition of substance which allows the reaction), and second, the context (the physical conditions which allow the mechanism to come into operation). This proposition that causal outcomes follow from mechanisms acting in contexts is the axiomatic base upon which all realist explanation builds (26, p. 58).

## Results and conclusion

Positivism places a wider knowledge of the world on observation and what can be developed and built systematically through research. CR emerged to address the strengths and weaknesses of positivism. What this means is that CR acknowledges the possibility of science but recognizes the social dimensions of humans and science and thereby resolves the difficulty usually encountered with positivism. CR takes the middle ground and does not reduce the world to a positivistic universal order, nor does it place objective truth-value on the perspectives of human beings or remove the influence and importance of human perspectives (23).

Clark et al. explain that researchers need to go beyond the surface of observable factors (the actual), to explore what is happening underneath (the real). This is because events in the actual domain are generated from complex interactions of factors in the real domain (23). For example, the results from the in-depth interview of smokers in Nigeria are mostly similar to what is out there in the academic discourse on smoking. Questions asked around why smokers smoked had similar responses to what is in the literature. Smokers’ smoke because smoking relaxes them, calms them, makes them think well/clearly, makes them happy, relieves depression and anxiety, and so on. One smoker said: ‘Yes, I stopped smoking for 2 months and I was not myself, that is, happy as I used to be, so I started again’. Concerning the reason why smokers started smoking, the smoker participants’ responses from Nigeria are in congruity to the scientific literature on smoking. Their responses include picking up smoking due to peer-pressure; due to family members smoking; due to the influence of tobacco company advertisements or due to tragic events happening in their lives (29–31).

Nonetheless, there were some differences in the Nigerian smoker participants’ responses and what is out there in the literature on smoking. This is based on the social and political environment of Nigeria. These differences are discussed as demonstrated through narratives from research participants. As an example, some of the participants attributed their tobacco addiction to something diabolic – to a spiritual problem. In this instance, the tobacco companies were not an issue, and the tobacco companies were not to blame for addiction to cigarettes/nicotine. Instead, the respondents stated the reason they could not stop smoking was attributed to a spiritual force greater than them and wanting to ruin them. In the words of one participant, ‘I think there is a spiritual force holding me back from stopping smoking, I have tried several times to stop smoking, but the spirit hinders it and my aunt suggested I go to a church so that they can pray for me for deliverance from smoking’. Another participant attributed his continuing to smoke to being under the hypnosis of a step mum who is trying to get him killed. ‘I think my step mum hates me and she is trying to kill me by putting me under some kind of spell so that I have no power to stop smoking, so that I can continue to smoke and die’.

In the West, smoking has been transformed from an accepted habit to an unacceptable one, and in some cases, a reviled habit. For example, in the United States, there is a National Youth Anti-Drug Media Campaign for youths to curb tobacco, called Live Above the Influence (32). It is apparent there is a perceptual shift in the West. However in Nigeria, many youths still believe it is cool and Western to smoke as demonstrated in the following narratives by research participants in this study; ‘I smoke because it makes me look cool, it makes me look like the whites, the Americans’. Another participant stated, ‘it was more of peer-pressure, I was trying to feel among (that is belong, be part of a group). That was back in my early days and formative years, you see your friends smoking and you try to belong and before I knew it I got addicted; Smoking gives some sense of superiority that is self-esteem (confidence). For instance when I'm talking to a girl, I will sound very dormant if I'm not smoking’. Another participant said, ‘If you want to look cool and civilized and you want to look American, then smoke; it is only uncivilized Nigerians who say smoking is bad’. These statements are understandable as there are no existing anti-smoking campaigns in Nigeria at the time this study was conducted. Therefore a shift in perception toward smoking has not occurred among the Nigerian youth as it has with the American youth.

Another issue that emerged from the study that is peculiar to Nigeria is the general cynicism and lack of trust toward the Nigerian government. The participants, health professionals, and smokers lack confidence in the Nigerian government to help the population fight the war against tobacco. The participants concluded that enacting smoke bans in Nigeria would not be fully supported by the Nigerian government because the government benefits financially from tobacco companies. For example, a research participant who is a tobacco control activist in Nigeria states that a senator who is on the pay list of a tobacco company, in the middle of a discussion to pass a tobacco bill in Nigeria, remarked that the tobacco bill will be passed over his dead body. The following narratives demonstrate the general cynicism expressed by participants toward the Nigerian government in terms of tobacco control in Nigeria:

They are not too serious about it (the Nigerian government) they need to be more serious

- Smoker participant

Firstly, the attitude of the Nigerian government is nothing to write home about, they are not behaving like people that actually want cigarette smoking to reduce, they are behaving like people that are collecting taxes and are even hoping that BAT (British American Tobacco) will make more money and get more taxes to pay

- Health professional

I think they are being politically correct; they don't want to say smoking is okay but they are acting like it's okay so I'm not sure we have the government on our side

- Health professional

And I think also that the federal government in Nigeria is a bit lax with the tobacco companies. The tobacco companies are having a big battle all over the world. They are coming to Nigeria and setting up big factories and claiming to be socially responsible by sponsoring different youth programs, and they are having an easy time here in Nigeria. So I think perhaps if the government was also strict on the tobacco company, tax the products, put heavy taxes on tobacco companies, very heavily so that the products come out very expensive right from production, I think that will help curb smoking in Nigeria

- Smoker participant

In addition, there is a lack of awareness about the health risks of smoking, and an underestimation of the risk, as demonstrated by the participants in the narratives below:

No, it not like I don't believe is bad (that is, smoking), that is why I said I have a brand which I feel to me are much better, that don't pose any health risk to me.

- Smoker participant

The major risk for me is smoking other kinds of cigarette except Benson. My uncle once advised me that smoking any other kind of cigarette could be harmful, one should stick to one brand of cigarette.

- Smoker participant

There seems to be a general belief among Nigerian smokers that smoking brand name cigarettes like Benson and Hedges makes smoking less harmful to their health. It is almost a thing of pride to mention the type of brands they smoke. It is believed that the more popular and expensive the brand, the less the adverse health effect. Another important point that came up in the course of the interviews is the mention of some local herbs that Nigerian smokers believe can be used to alleviate the adverse health effects of smoking. The belief is that such a herb helps to clean their lungs and heart of all the toxins from the cigarette smoked. This is demonstrated in the narratives below:

I take native drugs (Ghana root and Ogogoro – a concentrated alcoholic drink) they said it cleans the heart. But there is something I observed in it any time I wake up, I urinate and the color of my urine is yellow. I'm a chain smoker; I smoke 2 to 3 packs.

- Smoker participant

One could only have cough, chest pain and could be taken care of, because as a smoker one need special drugs that are good for smoker. For example, salt and water, sodium water, washes (clears) the throat and the chest.

- Smoker participant

But my friend told me about this bitter kola, that it's good and washes the heart, I used it and it was working, so I continue smoking.

- Smoker participant

For some smokers, the solution is a total ban on cigarette consumption as shown below:

Also people should stop selling fake cigarette.

- Smoker participant

I don't even think about the risk, you know 95% of smokers don't read the advert on the label if they buy cigarette.

- Smoker participant

I think I can quit if I wanted to but I'm not okay with the fact that Federal Ministry of Health have not done what they are supposed to, like ban the tobacco company or ask them to reduce the quantity of nicotine in the cigarette since they are not okay with the fact that people smoke.

- Smoker participant

The major risk is cancer of the lungs and this saying that smokers are liable to die young. So why are they still selling it since it has health implication.

- Smoker participant

Many groups have called for the prohibition of tobacco products to eradicate tobacco use entirely in society; however the prohibition of tobacco is unlikely to be either feasible or effective for a number of reasons. First, tobacco is a global product that will be hard to prohibit, for even when substances are prohibited, they continue to be widely used, as is the case with many illicit drugs. Second, prohibition creates its own sets of problems like smuggling: it is likely to increase criminal activity and entail costly police enforcement (33). Third, the prohibition of tobacco is unlikely to be politically acceptable in most countries. In India, recent attempts to ban a chewed type of tobacco known as gutka failed largely for political reasons (34). Fourth, from an economic (as opposed to public health) perspective, optimal consumption is not zero, given that some fully informed adults would still be interested in smoking (35). Fifth, drastic supply reductions would lead to significant welfare losses for the poorest farmers in countries like Africa highly dependent on tobacco as a source of cash income (33).

Finally, there could be unintended consequences for society when people who have been highly dependent on nicotine now suddenly find out that they no longer have access to it (36). Nicotine is highly addictive. Almost any time, a participant listed the benefits of smoking, it included one or more benefits that are the direct result of nicotine use, such as ‘if I have to do something stressful, I smoke’ or smoking is ‘relaxing’, smoking ‘helps me work’ – these are statements made in various forms, by every participant.

Another issue is from the perspective of female smokers and how they believe they deal with smoking risks. According to a female smoker, getting married and pregnancy are the only events that would produce an impetus to quit smoking ‘If I get married or I'm pregnant I will stop smoking’. Some other smokers also believe that the fewer the number of cigarettes they smoke, the better it is for them to reduce the health risks from smoking. According to one of the participants, ‘Whenever I start to have chest pain, I reduce the number of sticks I smoke’.

Below are some narratives from health professionals on risks and awareness of smoking in Nigeria:

We don't have enough information and the reason is that there is no awareness and advertisements are very expensive in Nigeria so this makes it difficult for a private organization to sponsor. Another thing is to begin to have culturally tailored messages maybe in local languages the way people will understand it. The person might not have seen one, because most advert are in specific magazines which poor smokers may not have access to, if we want to reach people on the street, advertisement should be more on radio that has wider coverage. A large proportion have access to radio, the advert must be culturally tailored like putting it in a local or pidgin language.

- Health professional

First of all, there is a low level of awareness of the specific risk involved in cigarette smoking in Nigeria, so it important that we continue to educate people, but aside from that we need to look at specific programs that pay attention to the real reason why people start and why they continue to smoke. Also peer-related intervention will also work and may be for the poorest of the poor, increasing the price could also work because right now cigarettes are still cheap.

- Health professional

So I think we should educate them, I'm not sure they are aware of the real dangers of cigarettes at the governmental level. And the reason why I'm saying they should be aware is that it's the beginning of it and from there they can have funds for tobacco campaign and so most of things we have as regards to anti-tobacco are just NGOs (Non- government Organizations). Like the way we have a concerted effort for HIV/AIDS, we don't have that for tobacco (cigarette smoking). So it will be nice to have increased awareness and increase on money spent on tobacco cessation activities and if possible a divorce from BAT (British American Tobacco).

- Health professional

First, education, that is informing everybody even people that have not started smoking. It is a youth-syndrome because of lack of education more youths go into smoking. When government sponsors a program that educate people (like HIV) this can help a lot.

- Health professional

Other issues that came up were the age of smokers and the cost of cigarettes. The cost of cigarette is so cheap, cheaper than confectionery, and so as a result, many young kids pick up smoking as early as age 9. This health professional explains; ‘Currently people as young as 9 year olds can afford to smoke because cigarette is very cheap, it is cheaper than tom-tom sweet. If I put N20 on my table I will not miss it because to me it is not a big deal; but if a child has N20 might buy more than five cigarettes but if we increase it to like N3,000 then it will discourage many people from buying it’.

A smoker echoed what many of the smokers in this study said about the age they picked up smoking; ‘When I was in primary school, I had people that smoke around, I was like 10 or 11 years old then, I had one brother that always send me around to buy and I will be watching him, there was this particular day that he dropped an unfinished cigarette because he had something to attend to, I just went to pick it up and that was how I started’.

Many of the participants also spoke on the effect of laws in Nigeria. There are many laws in Nigeria but because of the political climate, many of the laws do not incur penalties, and therefore the Nigerian population has total disregard for the laws. The participants described it this way:

I think we have that problem generally in Nigeria, we have some good things on paper but they are not implemented just like you would say cigarette shouldn't be sold to people less than 18 years. In a study I did, over 30% of children who were in secondary school have been sent to buy cigarette by other people and 9% of those that has been sent actually went and bought cigarettes themselves to smoke, and there is supposed to be a law that says a young person can't buy cigarette. I'm, not sure people are aware of the laws. And second of all I'm not sure if these laws are implemented, I think what works in Nigeria regarding implementation of law is when there is a penalty to be paid for breaking the law; then people will obey the law.

- Health professional

Increasing taxes on cigarettes has decreased smoking prevalence in the West, an example being the United States and Canada, where increasing taxes led to a reduction in smoking (1). Taxation may be an important mechanism for reducing the prevalence of smoking in developed countries, like the United States and Canada, this mechanism may be different in some countries in Africa. Even though higher taxes have translated to less people smoking, higher taxes may also have unintended consequences in some developing countries. For instance, in Nigeria, higher taxes on cigarettes may lead to cigarette smuggling, which will likely mean cigarettes will be sold at cheap prices. Many smokers may prefer to buy the less expensive cigarettes to the extent that this contraband may completely take over the tobacco market in a developing continent, such as Africa.

A study done in the United States by Halpen and Warner (1993) found that cost was not associated with decreased success in smoking cessation. They found little difference in the effect of cost as a reason for smoking cessation across demographic characteristics, including gender, age, education, minority status, and income. The authors gave a number of reasons why this might be so: maybe smokers react to price increases without consciously identifying them as a motivation to quit; or maybe tax increases discourage younger individuals from starting to smoke. In light of this study, it is recommended that more research needs to be done on the effectiveness of increasing taxes on cigarettes as an effective strategy for smoking cessation (30).

Historically, in developed countries, higher taxes on cigarettes translate to higher government revenues, which are sometimes used to improve population health. In Nigeria, increasing taxes on cigarettes may be ineffective, if the necessary legislations and infrastructures are not in place for the taxes to be earmarked as revenue for population health. In this context, placing higher taxes on cigarettes will quite literally mean taking money from the poor to give to the rich.

Another problem that can exacerbate the problem of smoking in Nigeria is poverty and people living in deprived neighborhoods. Living in a deprived neighborhood that suffers high crime rates, stress from poverty, limited opportunities for recreation, and an unpleasant physical environment is actually known to increase the prevalence of smoking (37), as some smokers indicated in the interviews:

‘I smoke because I don't have a job and I am frustrated’. I have a Master's degree and graduated 9 years ago and yet don't have a job, so I just smoke away my sorrows, especially when I see other people my age, who have jobs and drive nice cars.

- Smoker participant

I can ban cigarette since it causes more harm than good. I think people smoke as a result of frustration. Therefore total ban of those products can help reduce smoking rate. I think the government needs to re-evaluate their priority (I mean good governance, good policy) working towards economic prosperity. Because lot of smokers are graduates who are unemployed and got frustrated because of the economic status of the country.

- Smoker participant

Responses framed within empowering smokers through education on the adverse health effect of smoking will include tailoring tobacco control policies toward the specific needs and experiences of different groups (38, 39). An educational campaign replete with messages that smoking is unhealthy is less expensive, will empower smokers in Nigeria, and is likely to have a greater impact on reducing smoking prevalence in a place like Nigeria. Many studies have shown education as the strongest predictor of both smoking prevalence and quitting (31, 40–42). Education by itself is not sufficient enough to reduce smoking prevalence and quitting – other predictors come in the mix to support smoking reduction.

In Nigeria, the culture is highly diverse in different regions of the country. Essentially, the tobacco control policy that would work in the urban parts of Lagos, Nigeria may not necessarily work in the rural parts. Aptly honing in on the point is an example of a discovery made in the course of this study. While interviewing smokers in Nigeria, it became apparent that smokers in the upper socio-economic class had more information on the health hazards of smoking than smokers in the lower socio-economic class. The reason for this discrepancy is probably that the former group travel abroad regularly and, therefore have easy access to not only information from the West, but also access to smoking-hazard information through international television programs and other means of communication. During the study, when questions were asked about the risks of cigarette smoking, people in the upper socio-economic class readily provided well-informed answers. However, when asked the same questions about the risks of smoking, people from the lower socio-economic class hesitated and, in most cases, admitted that they will die from smoking. This common deduction appeared to be based on cigarette package warnings stating that smokers are liable to die from cigarette smoking.

## Concluding comments

The generative theory usually associated with CR ‘sees causation as acting internally as well as externally’. According to Pawson and Tilley (1997), ‘cause describes the transformative potential of phenomena. One happening may well trigger another but only if it is in the right condition in the right circumstances. Unless explanation penetrates to these real underlying levels, it is deemed to be incomplete’ (27, p. 34). CR's approach to smoking and tobacco control will be based on recommendations for research, context, and mechanism. In considering underlying mechanisms associated with smoking in Nigeria for example, using CR terms, accurate information was obtained from the grassroots (those affected directly by the policy) in order to obtain a clearer understanding of local problems of health and smoking.

During the study, it was discovered that some participants attributed their addiction to smoking to diabolism and blamed themselves or someone else for their addictive behavior – the blame was never on the tobacco companies. In this situation, a policy that would help combat smoking in Nigeria will have to harness some of these cultural beliefs in order for the policy to be effective for that particular population. Essentially that was what this study set out to do – to conduct on-site research (critical ethnography) to identify factors that hinder health and smoking regulation, bring to the fore some issues unique to Nigeria and capture attributes of a successful tobacco control policy in Nigeria.

A successful tobacco control policy based on CR's term will be based on tailoring tobacco policies to peculiarities of different population. The research objective was achieved through fieldwork, critical ethnography in Nigeria, interviewing Nigerian smokers and those in tobacco control policy in Nigeria (those that will be directly affected by the policy), and data was presented that can be translated into effective tobacco control policy specific to Lagos, Nigeria. The main point of this paper is that to be effective, tobacco programs or policies should be responsive to the particular national and socio-cultural contexts in which they are placed. CR theory is an appropriate and innovative approach with which to examine the tobacco problem and research in different resource settings, and to guide the formative research necessary to underpin the development of appropriate policies and interventions.

CR is a wider attempt aimed at harnessing the strengths and addressing the weaknesses of positivism, idealism, and relativism. What this means is that CR acknowledges the possibility of science but recognizes the social dimensions of humans and science in a manner that does not fall into problematic versions of relativism or positivism (29). Understanding how contexts (such as social and physical environments) and characteristics of the individual (such as age, race, and sex) interact to influence health appears to be an important step in designing effective health policies. Smoking is complex and likely to be affected by a number of factors. CR-driven research directs researchers to understand ‘what works for whom, when, and why and explore the complex ways in which interventions interact with people, professionals, and settings to cause different outcomes’ (27).

To this end, interventions must be localized and be responsive to particular places, societies, and the stakeholder's need for programs and policies to address contingencies and context-specific details that must be attended to in programs and interventions in different settings. The answer to whether CR produces a better approach for tobacco prevention and control is not known at this point as this is outside the scope of this study. However, the logic of CR theory is its attention to social and structural mechanisms, and the findings from this small ethnographic study suggest that CR holds promise for addressing underlying mechanisms that link complex influences on smoking. Different countries battle with different challenges socially, economically, and politically. For example, insulin, a drug commonly used to treat diabetes, in the Western world requires refrigeration. However, in resource-poor settings like some African countries, power or electricity is not stable/limited or not just available. In this instance, insulin cannot be considered as the best option in treating diabetes in resource-poor settings, because the environment does not support it due to lack of infrastructure. A lack of this important knowledge will undermine efforts at treating diabetes in resource-poor settings.

This paper espouses CR ideals and is a small example of a much broader re-evaluation of how health policies can be formulated and implemented with an emphasis on surrendering to context. The entire study is framed within this paradigm (surrendering to context) and has the potential to inform the knowledge base of global tobacco epidemic. It is inherent from conducting a critical ethnographic study in Nigeria that some issues were unique to Nigeria, for example, smokers attributing nicotine addiction to diabolic and spiritual problems; attributing smoking to Westernization and the underestimation of the adverse health effect of smoking; general cynicism of the public to the government; the belief that smoking a particular brand of cigarette reduces risk from smoking due to lack of knowledge on health and smoking; belief that some local herbs can protect smokers from the risks of cigarette smoking and the fact that kids as young as 9 years can buy cigarettes because there is no law/regulation in place that bans minors from purchasing cigarettes. The study also demonstrates that the rich in Nigeria have access to information that the poor do not. This shows further evidence to potentially understanding contexts prior to policy implementation.

This paper has raised an awareness that has the potential of birthing conscious effort that not only prevents us from travelling the same route, but also inspires us to develop effective and successful implementations on health and smoking in the 21st century. Further research to assess CR's utility in tobacco control is needed. Below are some recommendations for the way forward in combating tobacco in Nigeria and some of the recommendations are predicated on the findings in this study:

1. Surrendering to context/community engagement – understanding the environment, the history and culture of a place or people before successful implementation of a policy. Engaging those that will be directly affected by the policy in order to understand what the underlying issues are and how to successfully navigate the problems encountered.
2. To tailor tobacco control policies to the peculiarities of the people that would be directly affected by the policy.
3. There is a need for effective public education campaigns against smoking in Nigeria. Many of the smokers interviewed had limited knowledge on the adverse health effects of smoking and highly underestimated the dangers of cigarette smoking.
4. Setting up a body to fight tobacco companies in Nigeria, not stigmatizing smokers but the tobacco industry. Since tobacco companies are foreign multinational companies based in Nigeria, many Nigerian youths associate the tobacco companies with Westernization, and this is one of the key selling strategies of tobacco companies in Nigeria. While a stronger regulatory environment may not be sufficient enough to affect smoking prevalence, this study clearly identifies that the tobacco industry has found a welcome environment for tobacco promotion because of Nigeria's lack of regulations. In addition, many Nigerian tobacco policy experts endorse a strong regulatory environment.
5. Keep track of tobacco-related diseases and deaths and prevalence of tobacco use in Nigeria.
6. Further research to assess Critical Realism's utility in tobacco control is needed.

## Appendix A

**Interview and Focus Group Questions**

1. What are some of the challenges you face by continuing to smoke?
2. Why do you smoke?
3. Why do you continue to smoke?
4. What is the benefit associated with smoking for you?
5. Why did you start smoking?
6. What role does smoking play in your life?
7. How did you feel about smoking before you began?
8. What do you feel about smoking now?
9. Do you think you could quit if you wanted to?
10. What do you think is the major risk for you in smoking?
11. Is it the smoke or nicotine?
12. How do you deal with the risks presented from smoking?
13. Do you think the benefit you derive from smoking is from the smoke, the habit, the ritual, or nicotine?
  **At this point participants will take a short smoke break**
14. What can make you stop smoking?
15. What do you think of a substitute that has everything a cigarette has but has little or zero adverse health effect? Would you switch?
16. What do you think of a substitute that has everything like a cigarette but without smoke? Would you switch?
17. Have you tried to quit smoking?
18. What was it like?
19. How long did you stop smoking for?
20. Why did you start smoking again?
21. What do you think about smoking cessation products?
22. Which ones have you used? Did it help?
23. Has your medical doctor ever had any discussion about smoking with you?
24. What would you like to see the government do with regards to smoking?
25. What one thing would you like to see the government or medical community do with regards to smoking?

## Appendix B

**In-depth interview questions for health professionals**

1. What do you think will work to reduce smoking in Nigeria?
2. What do you think about anti-tobacco advertisements?
3. What do you think about regulations on tobacco?
4. What do you think about smoking cessation products? Why do you think that?
5. What, if any, help do you think you will need from the government or society to help smokers’ quit?
6. If you were to try to make smokers quit, what approach would you use (how would you go about doing it)?
7. What do you think about specific ads or messages to get smokers to quit
8. What specific government regulations/aids do you think would help smokers quit easily?
9. What is the government's position on smoking right now? How do you feel about that?
10. What do you think the government's position on smoking should be?
11. What would you like to see the government do in regards to smoking?

## Appendix C

**Smoking behavior background information sheet (this sheet was distributed to participants to fill shortly before the interview/focus group session starts)**

1. How often do you smoke cigarettes a day? ______________________
2. How often do you want to smoke in a day? ______________________
3. How many cigarettes do you smoke in a day? ____________________
4. When did you start smoking? _________________________________
5. How many years have you been smoking? _______________________
6. Where/When do you find you smoke the most? ___________________
7. Why do you think that is? _____________________________________
8. Are there certain situations that make you smoke? _________________
9. What situations? _____________________________________________
10. Do you think there is a pattern to your smoking (a schedule)? __________
11. How many people in your immediate family smoke? __________________
12. How many friends do you have who smoke? _________________________

**Thank you for your time!**

## Appendix D

(Questions used by Dunsi Oladele and the research assistant in Nigeria to recruit/select participants for the focus group questions and interviews).

1. Participants selected must respond ‘NO’ to each of the following
  - ‘I am currently in a quit-smoking program’
  - ‘I am currently using smoking cessation aids (such as nicotine patches or gum, or prescription anti-smoking drugs)’
  - ‘I am currently trying hard to quit smoking’
  - ‘I expect to quit smoking within the next year’
2. We will read several age categories and ask that participants tell us when we read the one into which their ages fall.
  18 – 34 1
  35 – 55 2
  RECORD EXACT AGE _______________________
3. We will ask for current level of education?
  Partial High School 1
  Completed High School 2
  Partial College / University 3
  Completed College / University 4
4. We will ask participants if they are able to participate in a 2 hour focus group session.
5. Respondents name: _______________________________________
  Phone number: ___________________________________________
  City: ____________________________________________________
  Email: ___________________________________________________
